# Radiation-irritated skin and hyperpigmentation may impact the quality of life of breast cancer patients after whole breast radiotherapy

**DOI:** 10.1186/s12885-021-08047-5

**Published:** 2021-03-31

**Authors:** Chin-Nan Chu, Kai-Chieh Hu, Rick Sai-Chuen Wu, Da-Tian Bau

**Affiliations:** 1 Graduatee Institute of Clinical Medical Science, China Medical University, Taichung City, Taiwan; 2 Department of Radiation Oncology, China Medical University Hospital, Taichung City, Taiwan; 3 Management Office for Health Data, China Medical University Hospital, Taichung City, Taiwan; 4 College of Medicine, China Medical University, Taichung City, Taiwan; 5 School of Medicine, China Medical University, Taichung City, Taiwan; 6 Department of Anesthesiology, China Medical University Hospital, Taichung City, Taiwan; 7 Graduate Institute of Biomedical Sciences, China Medical University, Taichung City, Taiwan; 8 Department of Medical Research, China Medical University Hospital, Taichung City, Taiwan

**Keywords:** Breast cancer, Radiotherapy, Quality of life, Sequelae, Dermatitis, Radiation-irritated skin

## Abstract

**Background:**

This study aimed to investigate skin condition, quality of life, and psychological impact of breast cancer patients after radiation therapy. We designed and administered a questionnaire to breast cancer survivors for better understanding the skin sequelae after radiation therapy.

**Methods:**

This study performed an anonymous online survey. Invitation join was posted in Facebook groups for Breast Cancer. Content of the questionnaire included basic information and a three-point scale on the degree of skin dryness, sweating, hotness sensation, itchy sensation, presence of pigment deposition, history of severe skin disorder, psychological impact, and quality of life after radiotherapy. Categorical variables were summarized using counts and percentages, and then Mantel–Haenszel chi-square tests, multiple correspondence analysis, Wald chi-square statistics, and logistic regression analyses were performed; *P* < 0.05 was considered statistically significant.

**Results:**

In total, 421 breast cancer survivors completed the questionnaire. Among them, 331 (78.62%) reported rarely sweating; 340 (80.76%) reported dry skin; 184 (43.71%) reported itchy skin in addition to dry skin; 336 (79.81%) had severe or mild skin color deposition; and 76 (18.05%) had eczema or contact dermatitis. Dry skin problems were caused by absent sweating and skin dryness in the irradiated skin area, post-RT severe skin disorders, and skin color deposition. Compared with patients sweating normally in the radiation field, patients with absent sweating and hotness sensation in the radiation field had a higher risk of depression.

**Conclusions:**

**This study showed that** breast cancer patients after whole breast radiotherapy may experience skin dryness, hypersensitivity and hyper pigmentation in the irradiated skin area. These “radiation-irritated skin” lesions may induce depressive psychological status and impact the quality of life in breast cancer patients after whole breast radiotherapy.

**Supplementary Information:**

The online version contains supplementary material available at 10.1186/s12885-021-08047-5.

## Introduction

According to global statistics, breast cancer is the most common female cancer. There are about 2.1 million newly diagnosed breast cancer cases in 2018, accounting for almost a quarter of female cancer cases [1]. Breast cancer therapy is a multi-disciplinary treatment with overall 5-year survival rate can be as high as 98 and 92% for stage I and stage II patients respectively [2].

Whole breast radiotherapy is an indispensable method in breast-conserving treatment as it decreases the rate of local recurrence significantly [3, 4]. Acute skin reaction is one of the most common adverse effects of radiation therapy. Redness, skin hotness, itching, edema, darkening, and desquamation(peeling of the skin), hyperpigmentation are its clinical features [5–7]. The severity of acute radiation dermatitis ranges from mild erythema to wet desquamation and even ulceration. In general, acute inflammation of the skin will gradually recover one to two weeks after radiotherapy and start re-epithelialization [8–11].

Chronic radiation dermatitis, which appears 90 days after the end of the course of treatment, may be permanent and irreversible. Common clinical manifestations include xerosis, hyperkeratosis, dyspigmentation, telangiectasia, and absent sweating [12–14]. Clinical observations and animal models indicate that the skin dryness and absent sweating is due to the pathophysiological changes of the sweat glands and sebaceous glands caused by radiation. In addition, increased epidermis thickness, keratinization, and subcutaneous collagen fiber loss were also reported after radiation exposure [12, 15].

Through our observation, skin after radiation therapy has skin dryness, pigmentation, and textural changes. Besides, irradiated skin will become more sensitive, easily irritated, prone to allergies. Some patients may even have symptoms of eczema and contact dermatitis on the irradiated skin area. Currently, there is no study on the effect of irritated skin on patients’ psychological impact and quality of life.

We designed a 3-point scaled anonymous questionnaire to investigate the skin condition, quality of life and psychological impact of breast cancer patients after receiving radiation therapy.

## Materials and methods

### Recruitment

This study is an anonymous online survey performed in the month of September 2019. Invitations to the survey, including a short introduction and a web-link to the questionnaire website, were posted in numerous private Facebook groups discussing breast cancer and accepting breast cancer patients. No financial reimbursement was offered for study participation. Once they agreed to be surveyed, volunteers were directed to this quality-of-life questionnaire. Ethical approval has been obtained from CMUH ethics committee (project ID number CMUH108-REC3–090).

### Questionnaire

This questionnaire was designed using the Google Forms. Participants only request to fill in their own email address, and no personal information was required. The basic information of the questionnaire included age, surgical procedures, chemotherapy, and interval after radiotherapy. Content of the questionnaire included a three-point scale on the degree of skin dryness, sweating, hotness sensation, itchy sensation, presence of pigment deposition, history of severe skin disorder, psychological impact and quality of life after radiotherapy.

### Statistical analysis

Because the study was an exploratory research, no sample size calculation was conducted.

Categorical variables were summarized using counts and percentages. Mantel-Haenszel chi-square tests were used to determine if there were linear associations between row variables and column variables in contingency tables when both were ordinal variables. A multiple correspondence analysis was adopted for dimension reduction in identifying associations among variables. The significance of explanatory variables in models was tested by Wald chi-square statistics. In logistic regression analyses, odds ratios were calculated to measure associations between explanatory variables and response variables. A *p*-value less than 0.05 was defined as a statistically significant difference. Statistical analyses were accomplished by SAS 9.4 (SAS Institute Inc., Cary, NC).

## Result

**Fig. 1:**
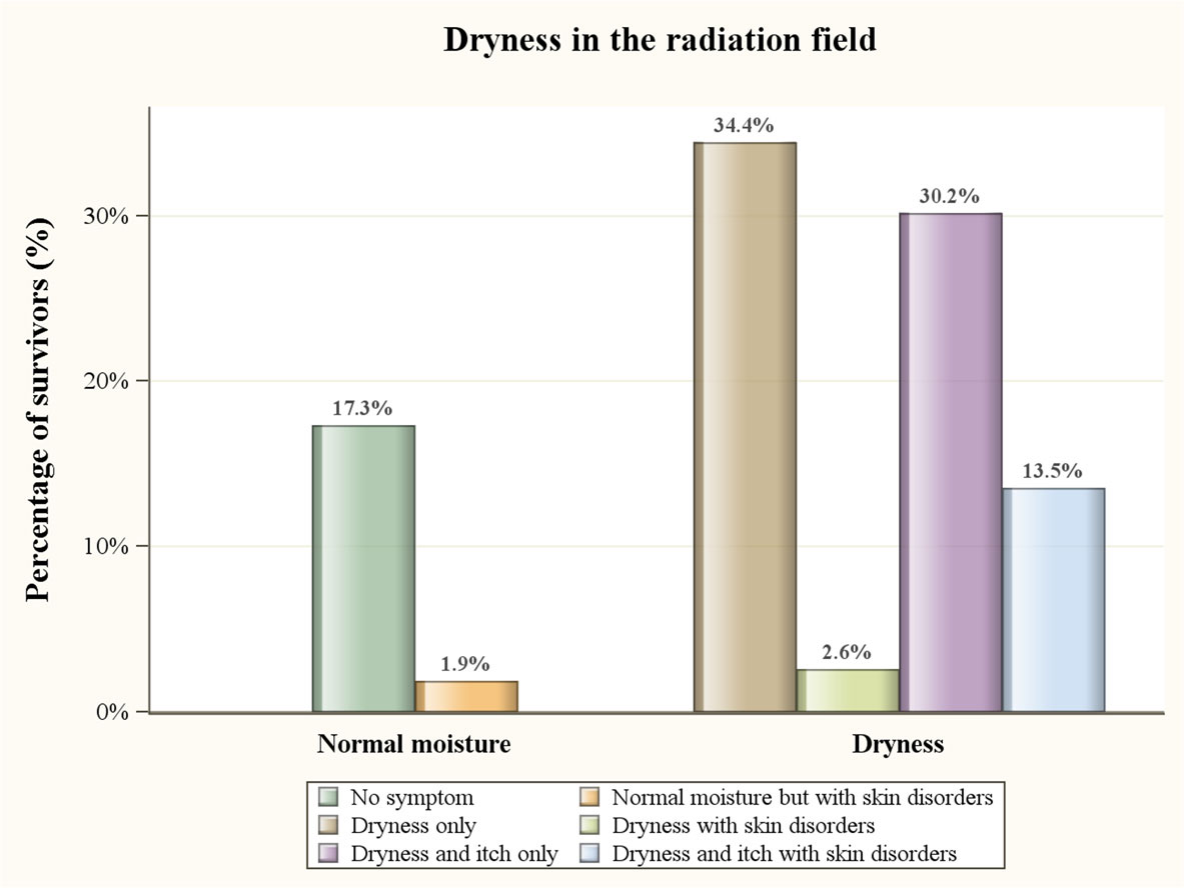
Distribution of survivors in the assessment of dryness status. 195 (46.32%) patients mentioned skin dryness combined with itching or some skin disorders (eczema or contact dermatitis), and 57 (13.54%) patients had dry skin, itching and skin disorder simultaneously.

Compliance rate was 100% for these 421 breast cancer survivors who completed the questionnaire. 333 (79.10%) patients were under 50 years old, and 337 (80.05%) received radiotherapy within two years. 331 (78.62%) patients reported absent sweating on the irradiated skin area, and 142(33.73%) patients reported absent sweating and hotness. 340 (80.76%) patients reported that their irradiated skin area was dry, and 184 (43.71%) patients reported that they suffered from dryness and itchy sensation on the irradiated skin area. It was reported that 336 (79.81%) patients had severe or mild skin color deposition and 76 (18.05%) patients had eczema or contact dermatitis. 89(21.14%) patients claimed to suffer from sadness or depression due to the skin problem (Table 1). In Figs. 1, 195 (46.32%) patients mentioned skin dryness combined with itching or some skin disorders (eczema or contact dermatitis), and 57 (13.54%) patients had dry skin, itching and skin disorder simultaneously.

**Table 1:** Characteristics of breast cancer survivors undergoing whole breast irradiation

Characteristics	Number of persons(***N*** = 421)	Percentage(%)
Age, years		
< 30	12	2.85
30–40	109	25.89
40–50	212	50.36
50–60	74	17.58
> = 60	14	3.33
Operation		
Total mastectomy	100	23.75
Partial mastectomy	321	76.25
Chemotherapy		
Yes	305	72.45
No	116	27.55
Interval after radiotherapy, months		
< 1	44	10.45
1–6	109	25.89
6–12	91	21.62
12–24	93	22.09
> 24	84	19.95
Sweating in the radiation field		
Absent sweating and hotness	142	33.73
Absent sweating	189	44.89
Normal	90	21.38
Dryness in the radiation field		
Dry skin and itchy	184	43.71
Dry skin	156	37.05
Normal	81	19.24
Seasonal skin irritation		
Yes	191	45.37
No	230	54.63
History of post-RT severe skin disorder (eczema, contact dermatitis)		
Yes	76	18.05
No	345	81.95
Skin color deposition		
Severe	112	26.60
Mild	224	53.21
None	85	20.19
Bother with dry skin		
Yes	211	50.12
No	210	49.88
Bother with skin color		
Yes	244	57.96
No	177	42.04
Ever felt sadness or depression due to the skin problem		
Yes	89	21.14
No	332	78.86

**Abbreviation: RT, Radiotherapy**

**Fig. 2:**
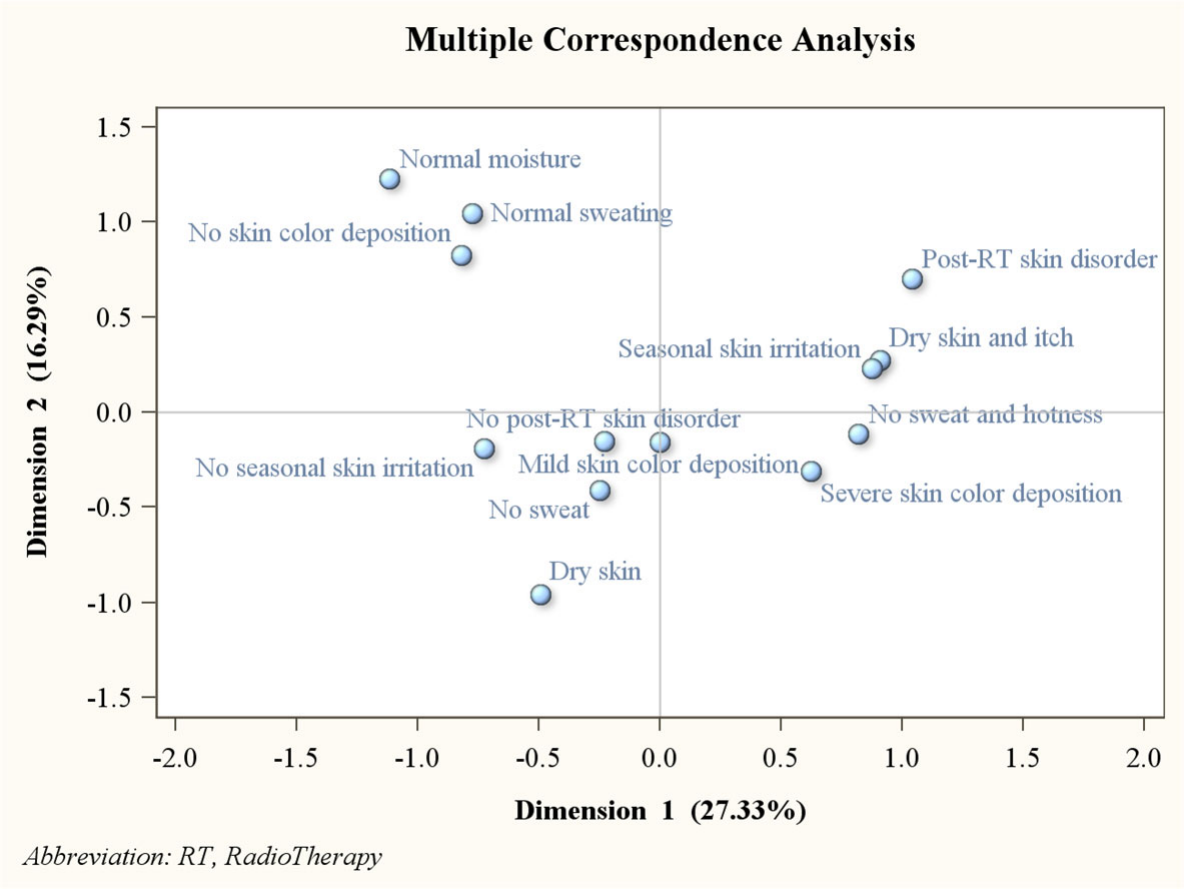
Multiple correspondence analysis of skin conditions. Dimension 1 is defined as the status of cuticle. Skin conditions got worse with increasing values in Dimension 1. Dimension 2 is defined as the function of sweat glands. Skin moisture got worse with decreasing values in Dimension 2.

Multiple correspondence analysis of skin conditions is displayed in Fig. 2, it was shown that 27.33% of the variance was explained by Dimension 1 and 16.29% of the variance was explained by Dimension 2. Skin conditions got worse with increasing values in Dimension 1. Thus, Dimension 1 is defined as ***the status of cuticle***. Skin moisture got worse with decreasing values in Dimension 2. Thus, Dimension 2 is defined as the ***function of sweat glands***.

In Table 2a, dry skin problems in the irradiated area could be caused by absent sweating (*p* = 0.0042), skin dryness (*p* < .0001), post-RT severe skin disorder (*p* = 0.0193), and skin color deposition (p < .0001). No significant difference was showed among different interval after radiotherapy. Compared to patients sweating normally in the irradiated area, patients with absent sweating and hotness sensation in the irradiated area had a higher risk of being bothered with dry skin (adjusted OR, 3.75 with a 95% CI, 1.71–8.21). Also, compared to patients sweating normally in the irradiated area, patients only with absent sweating in the radiation had a higher risk of being bothered with dry skin (adjusted OR, 2.40 with a 95% CI, 1.16–4.96). In Table 2b, depression due to the skin problems is highly correlated with absent sweating (*p* = 0.0002) and skin color deposition in the irradiated area (*p* = 0.0008), and weakly correlated with interval after radiotherapy (*p* = 0.0446). Skin color deposition had significant difference in interval after radiotherapy (*p* < 0.0001). There is no significant difference in interval after radiotherapy with sweating, dryness, seasonal skin irritation, and history of post-RT severe skin disorder. (Supplementary S 1–5).

**Table 2:** **Effects of baseline characteristics and skin conditions bothered by skin symptoms and signs in patients**

Factor	Yes	No	p-value^*#^
**a**			
**Bother of dry skin**			
**Sweating in the radiation field**			0.0042*
Absent sweating and hotness	102 (71.83)	40 (28.17)	
Absent sweating	89 (47.09)	100 (52.91)	
Normal	20 (22.22)	70 (77.78)	
**Dryness in the radiation field**			<.0001*
Dry skin and itchy	142 (77.17)	42 (22.83)	
Dry skin	66 (42.31)	90 (57.69)	
Normal	3 (3.70)	78 (96.30)	
**Seasonal skin irritation**			0.0796
Yes	138 (72.25)	53 (27.75)	
No	73 (31.74)	157 (68.26)	
**History of post-RT severe skin disorder**			0.0193*
Yes	57 (75.00)	19 (25.00)	
No	154 (44.64)	191 (55.36)	
**Skin color deposition**			<.0001*
Severe	82 (73.21)	30 (26.79)	
Mild	111 (49.55)	113 (50.45)	
None	18 (21.18)	67 (78.82)	
**Interval after radiotherapy, months**			0.1609
< 1	22 (50.00)	22 (50.00)	
1–6	53 (48.62)	56 (51.38)	
6–12	53 (58.24)	38 (41.76)	
12–24	48 (51.61)	45 (48.39)	
> 24	35 (41.67)	49 (58.33)	
**b**			
**Depression due to the skin problems**			
**Interval after radiotherapy, months**			0.0446*
< 1	7 (15.91)	37 (84.09)	
1–6	21 (19.27)	88 (80.73)	
6–12	19 (20.88)	72 (79.12)	
12–24	24 (25.81)	69 (74.19)	
> 24	18 (21.43)	66 (78.57)	
**Sweating in the radiation field**			0.0002*
Absent sweating and hotness	52 (36.62)	90 (63.38)	
Absent sweating	29 (15.34)	160 (84.66)	
Normal	8 (8.89)	82 (91.11)	
**Dryness in the radiation field**			0.1349
Dry skin and itch	48 (26.09)	136 (73.91)	
Dry skin	35 (22.44)	121 (77.56)	
Normal	6 (7.41)	75 (92.59)	
**Skin color deposition**			0.0008*
Severe	38 (33.93)	74 (66.07)	
Mild	47 (20.98)	177 (79.02)	
None	4 (4.71)	81 (95.29)	

**Abbreviation: RT, Radiotherapy**

^*^: A p-value less than 0.05 indicates that the factor affects the bother of skin symptoms

^**#**^**: Wald chi-square test**

^*^: A ***p*-value less than 0.05 indicates that the factor affects the bother of skin symptoms**

^**#**^**: Wald chi-square test**

Compared to patients sweating normally in the irradiated area, patients with absent sweating and hotness sensation in the irradiated area had a higher risk of getting depression (adjusted OR, 4.13 with a 95% CI, 1.67–10.20). Therefore, patients with severe skin color deposition had a higher risk of getting depression in contrast to patients without skin color deposition (adjusted OR, 8.30 with a 95% CI, 2.61–26.42). Also, patients with mild skin color deposition had a higher risk of getting depression in contrast to patients without skin color deposition (adjusted OR, 4.26 with a 95% CI, 1.41–12.83). Under multivariable analysis, here is no significant between interval after radiotherapy and depression due to the skin problem. (Table 3).

**Table 3:** Odds ratios of patients bothered by skin symptoms for factors in baseline characteristics and skin conditions

Depression due to the skin problems		
	Crude	Adjusted^*#^
**Factor**	**OR (95% CI)**	**OR (95% CI)**
**Age, years**		
< 30	4.29 (0.65, 28.26)	7.84 (0.98, 62.69)
30–40	1.43 (0.30, 6.89)	2.00 (0.35, 11.53)
40–50	1.85 (0.40, 8.55)	2.35 (0.43, 12.74)
50–60	1.05 (0.21, 5.34)	1.13 (0.19, 6.77)
> = 60	1 (Reference)	1 (Reference)
**Sweating in the radiation field**		
Normal sweating	1 (Reference)	1 (Reference)
Absent sweating	1.86 (0.81, 4.24)	1.36 (0.55, 3.33)
Absent sweating and hotness	5.92 (2.65, 13.20)	4.13* (1.67, 10.20)
**Dryness in the radiation field**		
Normal moisture	1 (Reference)	1 (Reference)
Dry skin	3.62 (1.45, 9.01)	2.42 (0.89, 6.61)
Dry skin and itchy	4.41 (1.80, 10.79)	1.44 (0.49, 4.27)
**Skin color deposition**		
No skin color deposition	1 (Reference)	1 (Reference)
Mild skin color deposition	5.38 (1.87, 15.43)	4.26* (1.41, 12.83)
Severe skin color deposition	10.40 (3.54, 30.54)	8.30* (2.61, 26.42)
**Interval after radiotherapy, months**		
< 1	0.69 (0.27, 1.81)	0.33* (0.11, 0.96)
1–6	0.88 (0.43, 1.77)	0.58 (0.26, 1.29)
6–12	0.97 (0.47, 2.00)	0.56 (0.24, 1.29)
12–24	1.28 (0.63, 2.56)	1.32 (0.59, 2.96)
> 24	1 (Reference)	1 (Reference)

^*^: When a 95% CI does not include the value of 1, it is indicated that there is a tendency among factor levels

^**#**^**: Multivariable analysis including age, operation, chemotherapy, interval after radiotherapy, sweating, dryness, seasonal skin irritation, post-RT skin disorder and skin color deposit**

## Discussion

To the best of our knowledge, this study is the first to conduct a quality-of-life questionnaire for skin problems after radiation therapy. Our data indicate that breast cancer patients undergoing whole breast radiotherapy may develop permanent less or no sweating of the irradiated skin area with the skin persistently dried, sensitive, and easily irritated. What is more, nearly one-fifth of patients suffer from recurrent skin disorders, such as eczema or contact dermatitis. Long-term skin pigmentation and irritated skin caused by breast radiation treatment negatively affects the physical and mental condition of breast cancer patients.

As we know, radiation dermatitis can be divided into acute and chronic radiation dermatitis depended by the time of occurrence. Acute radiation dermatitis, defined as a skin reaction that appears within 90 days of initiation of radiation exposure. There skin reactions usually start to occur within days to weeks after the initiation of radiation therapy [14]. The clinical manifestations are acute redness, edema, dyspigmentation, hair loss, and dry desquamation. In more severe cases, blisters, wet desquamation, or ulcers may occur [16–18]. Acute dermatitis usually recovers gradually within one to two weeks after the completion of radiotherapy. Chronic radiation dermatitis is defined as a skin reaction that appears more than 90 days after the end of radiation treatment [19]. The manifestations include epidermal thickness, dermis atrophy, keratinization, skin fibrosis, vascular injury, and telangiectasias [6, 12, 14]. Most of these chronic radiation dermatitis reactions are irreversible.

Ionizing radiation attacks the DNA of cells through the formation of free radicals, which damages the DNA double strand and makes the cells unable to undergo normal cell mitosis [20]. Early responding normal tissues, such as skin, mucosa and intestinal epithelium, are rapidly proliferative tissues and sensitive to radiation. Similarly, skin accessory tissues such as sweat glands, sebaceous glands, and hair follicles are also sensitive to radiation. In rat model, irradiation to the skin will result in irreversible pathological changes such as sebaceous glands loss, hair follicles loss, increased epidermal thickness, and skin fibrosis [6, 15]. We found skin dryness and hotness in the radiation field impacted the quality of life of breast survivors after radiotherapy and led to inferiority or depression. (adjusted OR, 4.13 with a 95% CI, 1.67–10.20).

It was reported that during radiotherapy, with the increase in the number of treatments and treatment doses, radiation skin reactions may impact the psychological status and quality of life of breast cancer patients [18]. Another patients-reported study indicated that 91% of patients after breast radiotherapy experienced hyperpigmentation, and 87.4 and 83.8% reported skin problems of skin dryness and skin roughness, respectively [16]. In our study, patients are very concerned about skin color deposition (*p* < .0001) and feel sadness or depressed about the hyperpigmentation. (Table 3) (adjusted OR, 8.30 with a 95% CI, 2.61–26.42).

**Fig. 3:**
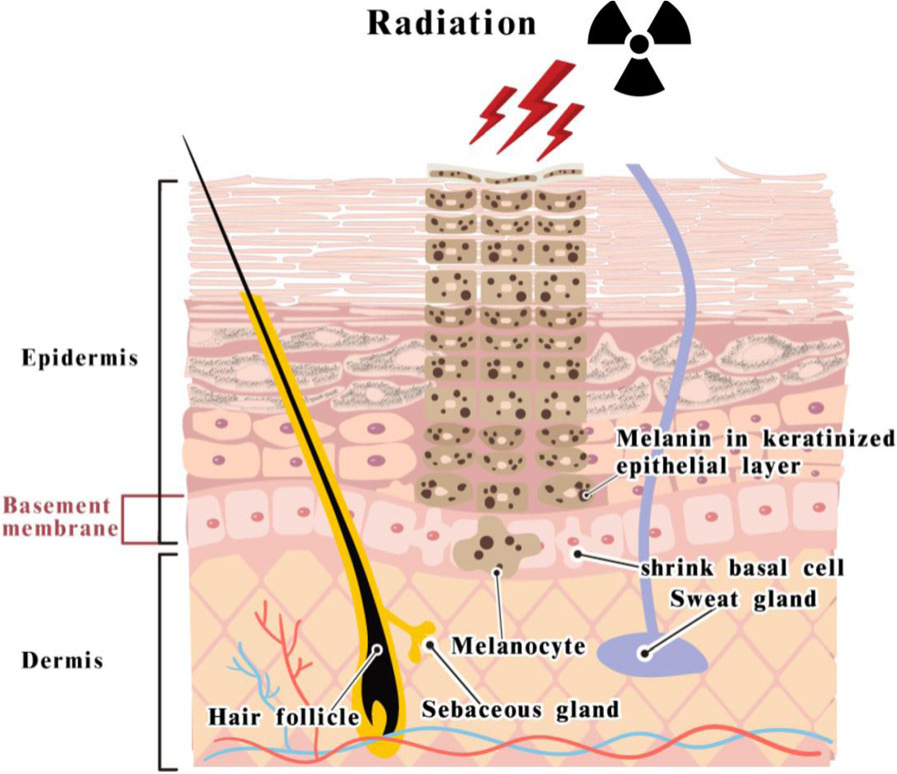
Possible pathological mechanism of “Radiation-irritated skin” and hyperpigmentation. Radiation may damage skin appendages, such as sweat gland, sebaceous gland, hair follicle and basement membrane. These damages may lead to the thickening of epidermis, atrophy of sweat gland and sebaceous gland, and hyperkeratosis. These may cause skin dryness and weakness of skin barrier. Radiation may also activate melanocytes, thereby promoting the formation of melanin and hyperpigmentation.

In addition to the lack of sweat and oil secretion in the irradiated skin area, 43.71% of patients reported skin itching, and 18.05% of patients experienced recurrent eczema or contact dermatitis on the skin after radiotherapy. We propose a new term, “Radiation-irritated skin”, defined as “Post-radiotherapy pruritic dry skin easily irritated by external stimulation, such as seasonal change or topical products”. “Radiation-irritated skin” is a brand-new concept of the sequelae of radiation therapy that no one reported in the past. According to previous studies, [6, 12, 14, 15] we concluded that the possible pathological mechanism of “Radiation-irritated skin” may consist of changes such as thickening of epidermis, hyperkeratosis, and atrophy of sweat gland and sebaceous gland.(Fig. 3) In this study, 46.3% of breast cancer patients after whole breast radiotherapy may develop “radiation-irritated skin”.

## Conclusion

This study showed that breast cancer patients after whole breast radiotherapy may experience skin dryness, hypersensitivity and hyper pigmentation in the irradiated skin area. These “radiation-irritated skin” lesions may induce depressive psychological status and impact the quality of life in breast cancer patients after whole breast radiotherapy.

Treatment of early breast cancer has achieved good results under current advanced medical technology. Better quality of life of breast cancer survivors should be a goal we pursue while we are treating these patients. We put forward the viewpoint of radiation-irritated skin, hoping to provide attention to skin care after radiation therapy.

## Supplementary Information

**Additional file 1.**

## Data Availability

All data generated or analyzed during this study are included in this published article.
