# Supplementary material for: Radiation-irritated skin and hyperpigmentation may impact the quality of life of breast cancer patients after whole breast radiotherapy
Source: BMC Cancer. 2021 Mar 31;21:330. doi: 10.1186/s12885-021-08047-5 (PMC8011219; doi:10.1186/s12885-021-08047-5)
Supplement: Supplementary file 1 — Additional file 1. [file 12885_2021_8047_MOESM1_ESM.pdf]

Radiation-irritated skin may impact the quality of life of breast cancer patients after whole breast radiotherapy

[1,2]Chin-Nan Chu, [3,4]Kai-Chieh Hu, [5,6,]*Rick Sai-Chuen Wu, [1,7,8,]*Da-Tian Bau

[1] Graduate Institute of Clinical Medical Science, China Medical University. [2] Department of Radiation Oncology, China Medical University Hospital. [3] Management office for Health Data, China Medical University Hospital. [4] College of Medicine, China Medical University. [5] School of Medicine, China Medical University. [6] Department of Anesthesiology, China Medical University Hospital. [7] Graduate Institute of Biomedical Sciences, China Medical University. [8] Department of Medical Research, China Medical University Hospital.

* Correspondence to rickwu@mail.cmuh.org.tw

[Corresponding Author Name & Email Address]

Prof. Rick Sai-Chuen Wu, M.D. and Prof. Da-Tian Bau, PhD equally contributed

rickwu@mail.cmuh.org.tw & artbau2@gmail.com

Table. S1

Sweating in the radiation field					
		No sweat and hotness n (%)	No sweat n (%)	Normal sweating n (%)	p-value[#]
Age, years	< 30	2 (16.67)	7 (58.33)	3 (25.00)	0.2995
	30-40	31 (28.44)	52 (47.71)	26 (23.85)	
	40-50	78 (36.79)	93 (43.87)	41 (19.34)	
	50-60	27 (36.49)	30 (40.54)	17 (22.97)	
	>= 60	4 (28.57)	7 (50.00)	3 (21.43)	
Operation	Total mastectomy	37 (37.00)	44 (44.00)	19 (19.00)	0.3774
	Partial mastectomy	105 (32.71)	145 (45.17)	71 (22.12)	
Chemotherapy	Yes	98 (32.13)	141 (46.23)	66 (21.64)	0.3985
	No	44 (37.93)	48 (41.38)	24 (20.69)	
Interval after radiotherapy, months	< 1	17 (38.64)	16 (36.36)	11 (25.00)	0.7614
	1-6	36 (33.03)	49 (44.95)	24 (22.02)	
	6-12	40 (43.96)	33 (36.26)	18 (19.78)	
	12-24	24 (25.81)	43 (46.24)	26 (27.96)	
	> 24	25 (29.76)	48 (57.14)	11 (13.10)	

[#]: Wald chi-square test.

Table. S2

Dryness in the radiation field					
		Dry skin and itch n (%)	Dry skin n (%)	Normal n (%)	p-value#
Age, years	< 30	5 (41.67)	5 (41.67)	2 (16.67)	0.9634
	30-40	41 (37.61)	41 (37.61)	27 (24.77)	
	40-50	106 (50.00)	72 (33.96)	34 (16.04)	
	50-60	28 (37.84)	33 (44.59)	13 (17.57)	
	>= 60	4 (28.57)	5 (35.71)	5 (35.71)	
Operation	Total mastectomy	39 (39.00)	42 (42.00)	19 (19.00)	0.4985
	Partial mastectomy	145 (45.17)	114 (35.51)	62 (19.31)	
Chemotherapy	Yes	132 (43.28)	118 (38.69)	55 (18.03)	0.7312
	No	52 (44.83)	38 (32.76)	26 (22.41)	
Interval after radiotherapy, months	< 1	23 (52.27)	15 (34.09)	6 (13.64)	0.1263
	1-6	47 (43.12)	39 (35.78)	23 (21.10)	
	6-12	43 (47.25)	35 (38.46)	13 (14.29)	
	12-24	41 (44.09)	32 (34.41)	20 (21.51)	
	> 24	30 (35.71)	35 (41.67)	19 (22.62)	

#: Wald chi-square test.

Table. S3

Seasonal skin irritation				
		**Yes n (%)**	**No n (%)**	**p-value#**
Age, years	< 30	8 (66.67)	4 (33.33)	0.8061
	30-40	42 (38.53)	67 (61.47)	
	40-50	103 (48.58)	109 (51.42)	
	50-60	30 (40.54)	44 (59.46)	
	>= 60	8 (57.14)	6 (42.86)	
Operation	Total mastectomy	41 (41.00)	59 (59.00)	0.3156
	Partial mastectomy	150 (46.73)	171 (53.27)	
Chemotherapy	Yes	138 (45.25)	167 (54.75)	0.9350
	No	53 (45.69)	63 (54.31)	
Interval after radiotherapy, months	< 1	22 (50.00)	22 (50.00)	0.6482
	1-6	41 (37.61)	68 (62.39)	
	6-12	52 (57.14)	39 (42.86)	
	12-24	44 (47.31)	49 (52.69)	
	> 24	32 (38.10)	52 (61.90)	

#: Wald chi-square test.

Table. S4

History of post-RT severe skin disorder (eczema, contact dermatitis)				
		Yes<br>n (%)	No<br>n (%)	p-value#
Age, years	< 30	2 (16.67)	10 (83.33)	0.1858
	30-40	13 (11.93)	96 (88.07)	
	40-50	45 (21.23)	167 (78.77)	
	50-60	12 (16.22)	62 (83.78)	
	>= 60	4 (28.57)	10 (71.43)	
Operation	Total mastectomy	15 (15.00)	85 (85.00)	0.3640
	Partial mastectomy	61 (19.00)	260 (81.00)	
Chemotherapy	Yes	54 (17.70)	251 (82.30)	0.7641
	No	22 (18.97)	94 (81.03)	
Interval after radiotherapy, months	< 1	8 (18.18)	36 (81.82)	0.9568
	1-6	19 (17.43)	90 (82.57)	
	6-12	19 (20.88)	72 (79.12)	
	12-24	14 (15.05)	79 (84.95)	
	> 24	16 (19.05)	68 (80.95)	

#: Wald chi-square test.

Table. S5

Skin color deposition					
		Severe n (%)	Mild n (%)	None n (%)	p-value[#*]
Age, years	< 30	5 (41.67)	6 (50.00)	1 (8.33)	0.9316
	30-40	24 (22.02)	60 (55.05)	25 (22.94)	
	40-50	59 (27.83)	115 (54.25)	38 (17.92)	
	50-60	19 (25.68)	36 (48.65)	19 (25.68)	
	>= 60	5 (35.71)	7 (50.00)	2 (14.29)	
Operation	Total mastectomy	26 (26.00)	52 (52.00)	22 (22.00)	0.6852
	Partial mastectomy	86 (26.79)	172 (53.58)	63 (19.63)	
Chemotherapy	Yes	81 (26.56)	155 (50.82)	69 (22.62)	0.2265
	No	31 (26.72)	69 (59.48)	16 (13.79)	
Interval after radiotherapy, months	< 1	21 (47.73)	21 (47.73)	2 (4.55)	<.0001*
	1-6	31 (28.44)	63 (57.80)	15 (13.76)	
	6-12	30 (32.97)	48 (52.75)	13 (14.29)	
	12-24	15 (16.13)	53 (56.99)	25 (26.88)	
	> 24	15 (17.86)	39 (46.43)	30 (35.71)	

[*]: A p-value less than 0.05 indicates that the factor affects the bother of skin symptoms.

[#]: Wald chi-square test.

Questionnaire for skin condition after whole breast radiotherapy

1. How old are you? _____________
2. What kind of breast surgery did you receive? □ Total mastectomy □ Partial mastectomy
3. Have you received chemotherapy? □ Yes □ None
4. How long have you been after radiotherapy? □ within one month □ 1~6 months □ 6-12 months □ 1~2 years □ more than 2 years
5. What is the sweating situation and temperature change of the skin after radiotherapy?
   □ Absent sweating and hotness □ Absent sweating □ Normal
6. Does the skin in the radiotherapy area feel dry or itchy?
   □ Dry skin and itchy □ Dry skin □ Normal
7. Have you ever felt seasonal skin discomfort in the radiotherapy area?
   □ Yes □ Few □ None
8. Have there been any skin disorder such as eczema, contact dermatitis on the skin of the radiotherapy area?
   □ Yes □ Never
9. Is there skin color change or deposition after radiotherapy
   □ Severe □ Mild □ None
10. Have you ever been troubled by skin problems after radiotherapy?
    □ Dry skin □ Skin color □ None
11. Have you ever felt sadness or depression due to skin problem after radiotherapy?
    □ Yes □ Few □ None
